# A Novel Manganese Efflux System, YebN, Is Required for Virulence by *Xanthomonas oryzae* pv. *oryzae*

**DOI:** 10.1371/journal.pone.0021983

**Published:** 2011-07-14

**Authors:** Chunxia Li, Jun Tao, Daqing Mao, Chaozu He

**Affiliations:** 1 State Key Laboratory of Plant Genomics, Institute of Microbiology, Chinese Academy of Sciences, Beijing, China; 2 Graduate School of Chinese Academy of Sciences, Beijing, China; 3 CAS Key Laboratory of Pathogenic Microbiology and Immunology, Institute of Microbiology, Chinese Academy of Sciences, Beijing, China; 4 School of Life Sciences, Tsinghua University, Beijing, China; 5 Hainan Key Laboratory for Sustainable Utilization of Tropical Bioresource, Hainan University, Haikou, Hainan, China; University of Massachusetts Medical School, United States of America

## Abstract

Manganese ions (Mn^2+^) play a crucial role in virulence and protection against oxidative stress in bacterial pathogens. Such pathogens appear to have evolved complex mechanisms for regulating Mn^2+^ uptake and efflux. Despite numerous studies on Mn^2+^ uptake, however, only one efflux system has been identified to date. Here, we report on a novel Mn^2+^ export system, YebN, in *Xanthomonas oryzae* pv. *oryzae* (*Xoo*), the causative agent of bacterial leaf blight. Compared with wild-type PXO99, the *yebN* mutant was highly sensitive to Mn^2+^ and accumulated high concentrations of intracellular manganese. In addition, we found that expression of *yebN* was positively regulated by Mn^2+^ and the Mn^2+^-dependent transcription regulator, MntR. Interestingly, the *yebN* mutant was more tolerant to methyl viologen and H_2_O_2_ in low Mn^2+^ medium than PXO99, but more sensitive in high Mn^2+^ medium, implying that YebN plays an important role in Mn^2+^ homoeostasis and detoxification of reactive oxygen species (ROS). Notably, deletion of *yebN* rendered *Xoo* sensitive to hypo-osmotic shock, suggesting that YebN may protect against such stress. That mutation of *yebN* substantially reduced the *Xoo* growth rate and lesion formation in rice implies that YebN could be involved in *Xoo* fitness in host. Although YebN has two DUF204 domains, it lacks homology to any known metal transporter. Hence, this is the first report of a novel metal export system that plays essential roles in hypo-osmotic and oxidative stress, and virulence. Our results lay the foundations for elucidating the complex and fascinating relationship between metal homeostasis and host-pathogen interactions.

## Introduction

The acquisition of transition metal ions is critical for normal cell metabolism and plays an important role in pathogen virulence [1]. Cells require a constant source of metal ions to conduct their regulatory, catalytic or physiological processes and imbalances can result in disorder or death [1]. In addition, excessive accumulation of certain metal ions can be toxic to the cell [2]. Hence, bacteria rely on highly specialized mechanisms to maintain intracellular metal homoeostasis. The transition metal Mn^2+^ ion is an important cofactor for a number of enzymes, contributes to protection against oxidative stress, and is required for virulence [1], [3], [4]. For example, the Mn^2+^-depedent enzyme, SodA, is involved in scavenging of reactive oxygen species (ROS) produced by the host and mutation of *sodA* results in decreased virulence in several species of bacteria, which implies that Mn^2+^ is important for bacterial pathogenicity [1].

Bacteria have evolved a number of sophisticated mechanisms to acquire manganese from their environment. Nramp H^+^-Mn^2+^ transporters and ATP-binding cassette (ABC) Mn^2+^ permeases comprise the principal manganese uptake systems utilized by most bacteria [1], [5], [6]. MntH, for example, a member of the Nramp family, is a proton-dependent divalent cation transporter originally described in eukaryotes and found in several bacterial species including *Escherichia coli*, *Brucella abortus* and *Salmonella* [3], [7]. Another example is SitABCD, an ABC-type transporter complex initially described as a Fe^2+^ transporter [5], [8]. Studies have also shown that some bacteria (i.e., *Lactobacillus*) appear to carry a third class of transporter, the Mn^2+^-transporting P-type ATPase [9]. In addition, BmtA (BB0219), a membrane protein with predicted homology to the Zn^2+^ GufA transporter family, has been identified as a manganese transporter in *Borrelia burgdorferi* [10]. With no homology to any known bacterial Mn^2+^ transporter, BmtA comprises a novel Mn^2+^ influx system.

Although cation efflux is also crucial for maintaining ion homoeostasis, very little is known about how manganese efflux is achieved. To date, only one bacterial Mn^2+^ efflux system (MntE) has been identified. MntE, a new member of the CDF family, functions as an Mn^2+^ efflux system in *Streptococcus pneumoniae* [11]. A mutant, designated Δ*mntE* has been identified that is sensitive to manganese stress, and MntE is required for virulence [11], [12]. Surprisingly, *mntE* does not have a homologue in all Gram-positive and Gram-negative bacteria. So just how Mn^2+^ efflux is achieved in bacteria lacking the MntE homologue remains unclear.

*Xanthomonas oryzae* pv. *oryzae* (*Xoo*), a Gram-negative bacterial pathogen, causes leaf blight, one of the most devastating diseases of rice worldwide. Although a number of virulence factors have been identified [13], [14], very little is known about the relationship between ion homoeostasis and virulence in *Xoo*. To date, only a few studies have shown that a breakdown in intercellular zinc or iron homoeostasis could cause impaired metabolism and virulence in *Xoo* [15], [16]. Because there is only one *mntH* homologue in the *Xoo* genome and our studies show that its expression is Mn^2+^ dependent (Figure S1), we speculate MntH mediates manganese influx. However, no manganese export system has been identified in *Xoo*, and MntE, the only known manganese exporter in bacteria, appears not to exist in this species.

In this study, we demonstrate a DUF204 domain containing protein, YebN, which is involved in manganese efflux and virulence in *Xoo*. We establish the relationship between oxidative stress and YebN. Also, we show that YebN can protect *Xoo* against hypotonic shock. Our findings highlight the importance of manganese homoeostasis in *Xoo* and should provide an example of a novel metal ion transporter family.

## Results

### YebN is an integral membrane protein with two conserved domains (DUF204)

**Figure 1:**
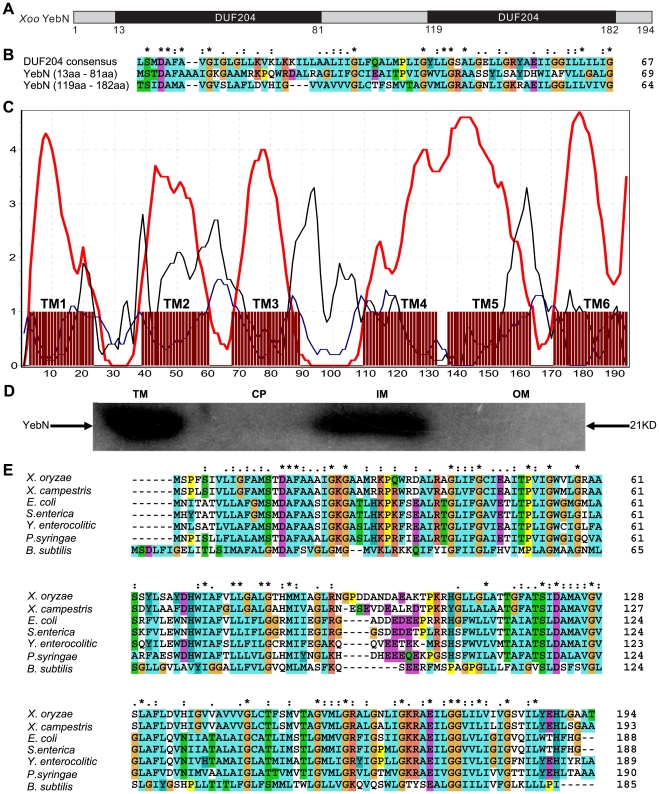
YebN is a conserved integral membrane protein with two DUF204 domains. (A) Domain organization of the *Xoo* YebN. Two DUF204 domains exist in YebN (http://pfam.sanger.ac.uk/family?acc=PF02659). (B) Alignment of two DUF204 domains (13–81 and 119–182) of YebN and the DUF204 conserved sequence. (C) Protein secondary structure was predicted by using Split-4.0 (http://split.pmfst.hr/split/4/). Red line: transmembrane helix preference; Blue line: beta preference; Black line: modified hydrophobic moment index; Maroon boxes (below abscisa): predicted transmembrane (TM) helix position. YebN contains six transmembrane (TM) helixes (TM1, TM2, TM3, TM4, TM5 and TM6). (D) A Western blot of *Xoo* cells (C-Δ*yebN*-His) total membrane proteins (TM), cytoplasmic fraction (CP), inner membrane fraction (IM) and outer membrane fraction (OM) was probed for subcellular location of YebN, using His tag antibodies. An equal amount (2 µg) of protein was loaded in each lane. The blot is representative of three independent experiments. (E) Sequence alignment of *Xoo* YebN against other bacterial homologues. *X*. *oryzae*: *Xanthomonas oryzae* pv. *oryzae* str. PXO99; *X*. *campestris*: *Xanthomonas campestris* pv. *campestris* str. 8004; *P*. *syringae*: *Pseudomonas syringae* pv. *tomato* str. DC3000; *E*. *coli*: *Escherichia coli* str. K-12 substr. MG1655; *B*. *subtilis*: *Bacillus subtilis* subsp. *subtilis* str. 168; *Y*. *enterocolitica*: *Yersinia enterocolitica* subsp. *enterocolitica* 8081; *S*. *enterica*: *Salmonella enterica* subsp. *enterica* serovar Heidelberg str. SL486. Alignments (B, E) were performed using ClustalW (http://www.ebi.ac.uk/Tools/msa/clustalw2/). The homology between the proteins (B, E) is indicated as follows: *, fully conserved residues; :, closed conservative substitutions; conservative substitutions.

We have created a mutant library of *Xoo* PXO99 by Tn5 insertion [17] and identified a virulence attenuated mutant (D6) whose interrupted DNA region comprised a 585 bp open reading frame (ORF). This ORF was identical to PXO_02753 in the *Xoo* PXO99^A^ strain and was annotated as *yebN* [18]. Bioinformatics analysis indicated that YebN contains two conserved domains with unknown function (DUF204) (Figure 1A and 1B). In addition, YebN was predicted to contain six transmembrane domains (Figure 1C). Western blots indicated that YebN was located at the integral membrane (Figure 1D). The location of this protein led us to postulate that it might function as a transporter. Moreover, bioinformatics analysis of the *yebN* promoter revealed a conserved MntR (a manganese dependent transcriptional regulator [7], [8]) binding site (Figure S2). Hence, we speculate YebN should function as a manganese transporter and conducted functional analysis of YebN in *Xoo*.

### YebN is involved in manganese export

**Figure 2:**
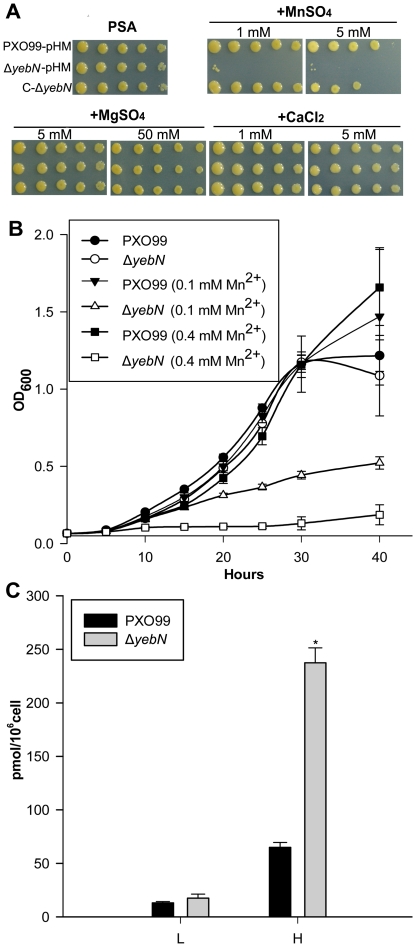
YebN is involved in manganese efflux in *Xoo*. (A) Phenotypes of wild type cells (*top row*), Δ*yebN* cells (*middle row*) and the complemented strain C-Δ*yebN* cells (*bottom row*) on PGA plates. Each spot was inoculated from 2 µl of a 10-fold dilution series from stationary cells (i.e., 10^0^, 10^−1^, 10^−2^, 10^−3^, and 10^−4^ fold from *left* to *right*). (B) The phenotypes observed from growth in liquid minimal medium and the effects of exogenous manganese in wild type and Δ*yebN* cells. (C) The cellular manganese content of the wild type and the *yebN* mutant grown in low- or high-manganese concentration. Cells were grown in media supplemented with 0 mM (L) or 0.15 mM MnSO_4_ (H). The manganese content of whole cells was determined by absorption spectroscopy. The data shown represents the mean ± standard deviations (SD) from four independent experiments (*P<0.01).

To investigate the YebN functions, we constructed the in-frame deletion mutant, Δ*yebN*, and a complemented strain, C-Δ*yebN* (see Materials and Methods). We tested Δ*yebN* growth in synthetic media (M4) supplemented with or without different nutrients including sugars, amino acids and metal ions, and found that only Mn^2+^ affected Δ*yebN* growth (data not shown). To confirm the Mn^2+^ phenotype, bacteria were cultured in rich medium (PSA) with or without Mn^2+^ and their growth monitored. Both the wild type and complemented strain grew well in medium supplemented with 1 mM Mn^2+^ in PSA medium (Figure 2A). Both strains also grew on plates supplemented with 5 mM Mn^2+^, albeit more slowly (Figure 2A). In contrast, Δ*yebN* did not grow on plates supplemented with either 1 or 5 mM Mn^2+^ (Figure 2A). We next examined the growth of these strains in liquid minimal medium (M4), measuring optical densities at 600 nm. The mutant showed a pronounced growth defect in Mn^2+^ supplemented medium (Figure 2B). Other ions including iron, copper, cobalt, nickel, cadmium and zinc had no different effects on PXO99 and Δ*yebN* growth (Figure S3). These data indicate that Δ*yebN* has increased sensitivity to manganese, relative to the wild type, suggesting a possible role for YebN in manganese export.

To confirm Δ*yebN* manganese export defect, we measured the intracellular levels of manganese in both PXO99 and Δ*yebN* using inductively coupled plasma mass spectrometry (ICP-MS). The *yebN* mutant accumulated four-fold more intracellular manganese than PXO99 when grown in medium containing 0.15 mM manganese (Figure 2C). There was no obvious difference in the intracellular manganese concentration between PXO99 and Δ*yebN* when grown in medium without manganese, however (Figure 2C). These results are consistent with YebN being a manganese exporter.

**Figure 3:**
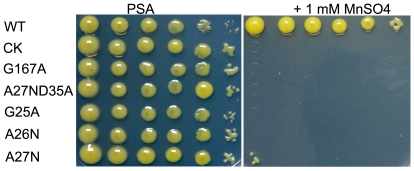
Amino acid substitutes in YebN cytoplasmic regions alter *Xoo* Mn^2+^ tolerance. The strains that *yebN* mutant (Δ*yebN*) carries extrachromosomal *yebN* wild type sequence (WT) or some point mutations in YebN cytoplasmic regions (G25A, A26N, A27N, A27ND35A and G167A) cloned into the pHM1 vector were analyzed as described in Figure 2A. The strain that Δ*yebN* contains the pHM1 vector was the negative control (CK).

To further study how YebN export Mn^2+^, we introduced point mutations into the cytoplasmic regions of the protein and identified which amino acids are critical for YebN function. We found that mutants G25A, A26N, A27N, G167A and A27ND35A all exhibit reduced growth in the presence of 1 mM manganese (Figure 3), indicating that these sites are functionally important. In contrast, mutation of the remaining cytoplasmic amino acids had no obvious effects on bacterial growth in media containing 1 mM manganese (Figure S4). Although we have yet to elucidate the exact function(s) of these particular sites, these results also implied that YebN is involved in Mn^2+^ efflux and provided a foundation for further studying the Mn^2+^ transport mechanisms.

**Figure 4:**
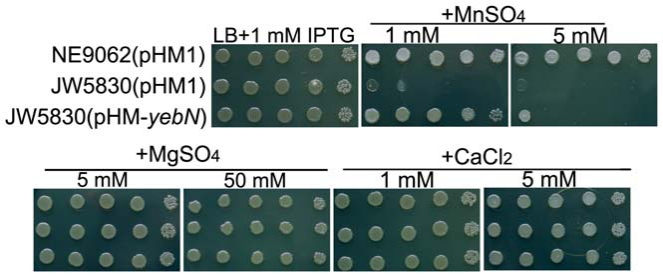
YebN functions in *Escherichia coli* are similar to those in *Xoo*. The sensitivity of *E*. *coli yebN* mutant (JW5830) to exogenous Mn^2+^ and the complementary analysis of JW5830 by the *Xoo* homologue. NE9062 (pHM1) and JW5830 (pHM1) are wild type *E*. *coli*. MG1655 and the *yebN* mutant harbor pHM1 plasmids, respectively. JW5830 (pHM-*yebN*) is a *yebN* mutant containing the *Xoo yebN* gene in a pHM1 vector. The experimental protocol was the same as described in Figure 2A, except bacteria were cultured in LB medium at 37°C.

Analysis of amino acid sequence alignments revealed that the *Xoo* YebN protein shared a high degree of sequence conservation with other bacteria such as *Pseudomonas syringae*, *Bacillus subtilis*, *Salmonella enterica* and *E*. *coli* (Figure 1E). Hence, YebN could be involved in manganese efflux in these species. We thus conducted the manganese toxicity assay in *E*. *coli* and found that the cells became sensitive to manganese stress when its *yebN* was mutated (strains JW5830) (Figure 4). Notably, this phenotype could be complemented by *Xoo yebN* (Figure 4). In addition, we found that JW5830 containing the *yebN* with amino acid substitution (G25A, A26N and G167A) that had not full functions in *Xoo* (Figure 3) also exhibited reduced growth in the presence of 1 mM manganese (Figure S4). These results demonstrate that YebN might function as a manganese exporter not just in *Xoo*, but also in other bacteria.

### Mn^2+^ up-regulates *yebN* expression

**Figure 5:**
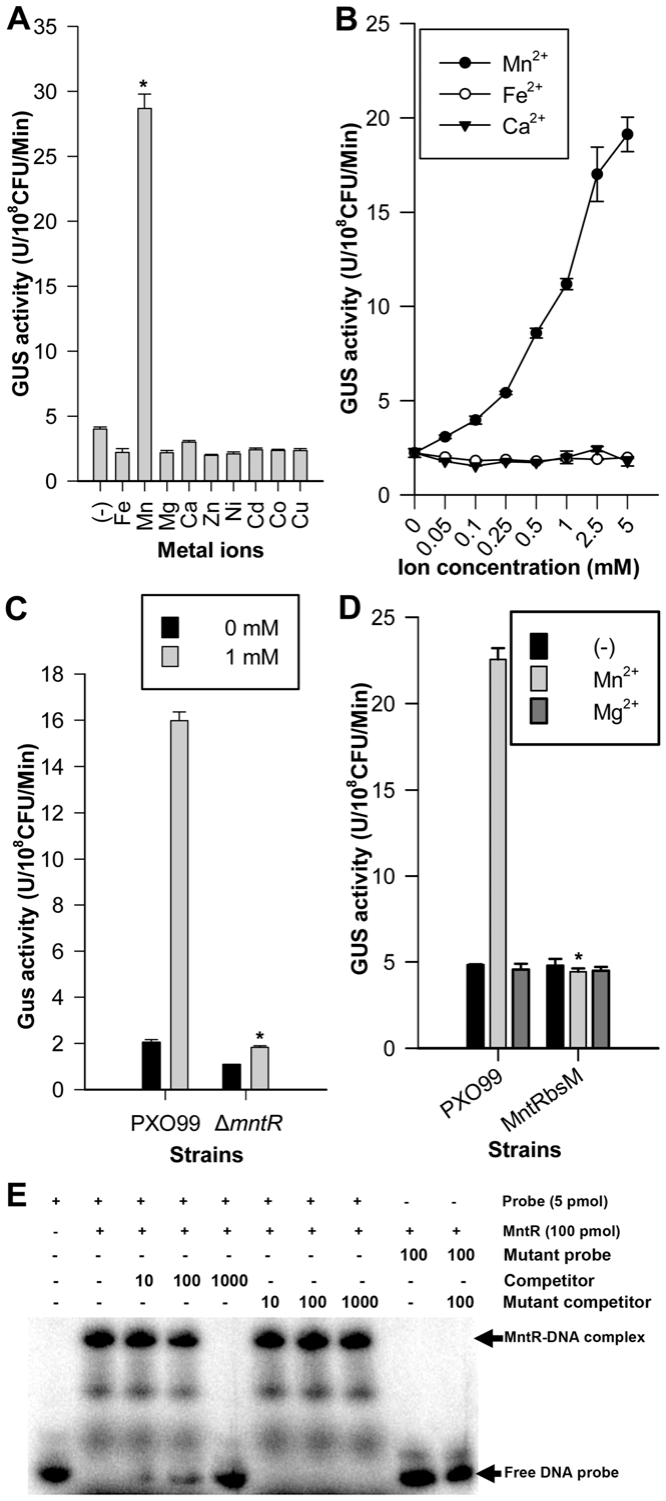
Mn^2+^ up-regulates the expression of *yebN* via MntR. (A) Wild type strains containing the *yebN* promoter *gusA* fusion were grown in M4 medium with or without the indicated ions. Cells were harvested by centrifugation and GUS activity was measured as described elsewhere [53]. (B) *yebN* expression (GUS activity) in wild type strains grown in PGA medium cantaining different concentration of Mn^2+^, Ca^2+^ or Fe^2+^. (C) The *yebN* expression in the *mntR* mutant in PSA medium cantaining different concentration of Mn^2+^ (*P<0.01). (D) The effect of the MntR binding site mutation in the *yebN* promoter on *yebN* expression. Error bars correspond to standard deviations (*P<0.01). (E) EMSA showing *in vitro* binding of MntR to the *yebN* promoter. His-tag *Xoo* MntR protein purified from *E. coli* BL21 (DE3) and the 95 bp ^32^P-labeled DNA fragment containing the predicted MntR binding site of the *yebN* promoter were used in the protein-binding assay. The ^32^P-labeled 95 bp DNA fragment containing the putative MntR binding site mutation was used as a mutant probe and the unlabeled fragment used as a mutant competitor.

The expression profiles of many metal ion transporters are regulated by their substrates [6], [7]. To investigate whether Mn^2+^ regulates *yebN* expression, the promoter was fused to the β-glucuronidase (*gusA*) gene and the construct ectopically integrated into *Xoo* genome by homologous recombination. To monitor *yebN* expression level, cells were cultured in M4 medium with various concentrations of divalent ions. Only Mn^2+^ increased *yebN* expression (Figure 5A). We also noted that GUS activity was enhanced in parallel with increases in Mn^2+^ concentration in the medium (Figure 5B). In addition, loss of YebN resulted in a 10-fold increase in its promoter activity without additional Mn^2+^ in medium (Figure S5), which also implies that Mn^2+^ regulates *yebN* expression because the *yebN* mutant could accumulate more intracellular manganese than PXO99(Figure 2C). Taken together, these data show that Mn^2+^ up-regulates *yebN* expression and YebN is involved in manganese homoeostasis in *Xoo*.

### MntR positively regulates the expression of *yebN*

Mn^2+^ down-regulates transporters such as *sitABCD* and *mntH* in many bacterial species via the transcriptional regulator MntR [5], [6]. Our results are consistent with this. Figure S1 shows that exogenous manganese negatively regulates *mntH* expression via the transcription factor MntR. Because Mn^2+^ regulates the expression of *yebN* and bioinformatics analysis of the *yebN* promoter revealed a conserved MntR binding site (Figure S2), we hypothesized that Mn^2+^ regulates *yebN* expression via MntR. To test this, we deleted the *mntR* gene from *Xoo* genome and introduced the *yebN* promoter-*gusA* fusion construct into it by homologous recombination. We examined the GUS activity in the wild type and *mntR* mutant grown in media containing 0 or 1 mM Mn^2+^. An increase of *yebN* expression was observed only in the presence of both Mn^2+^ and MntR (Figure 5C). Furthermore, the transcriptional activity of the *yebN* promoter was abolished, even in the presence of both Mn^2+^ and MntR, after the MntR binding site was mutated (*MntRbsM*) (Figure 5D and Figure S2). These findings show that in the presence of Mn^2+^, MntR up-regulated the expression of *yebN*, but down-regulated *mntH*.

To test whether MntR regulates *yebN* by directly binding to the promoter region, electrophoretic mobility shift assays (EMSA) were performed. MntR and the wild type probes formed a protein-DNA complex that migrated more slowly than the free probe. However, the signal from the DNA-protein complex gradually decreased when increasing amounts of the un-labeled promoter fragment were added (Figure 5F). In contrast, the mutant probe did not bind to MntR, and the un-labeled mutant promoter fragment did not affect formation of the DNA-protein complex (Figure 5F). These results are consistent with MntR binding to the *yebN* promoter and regulating its expression.

### YebN is important for protecting *Xoo* against oxidative stress

**Figure 6:**
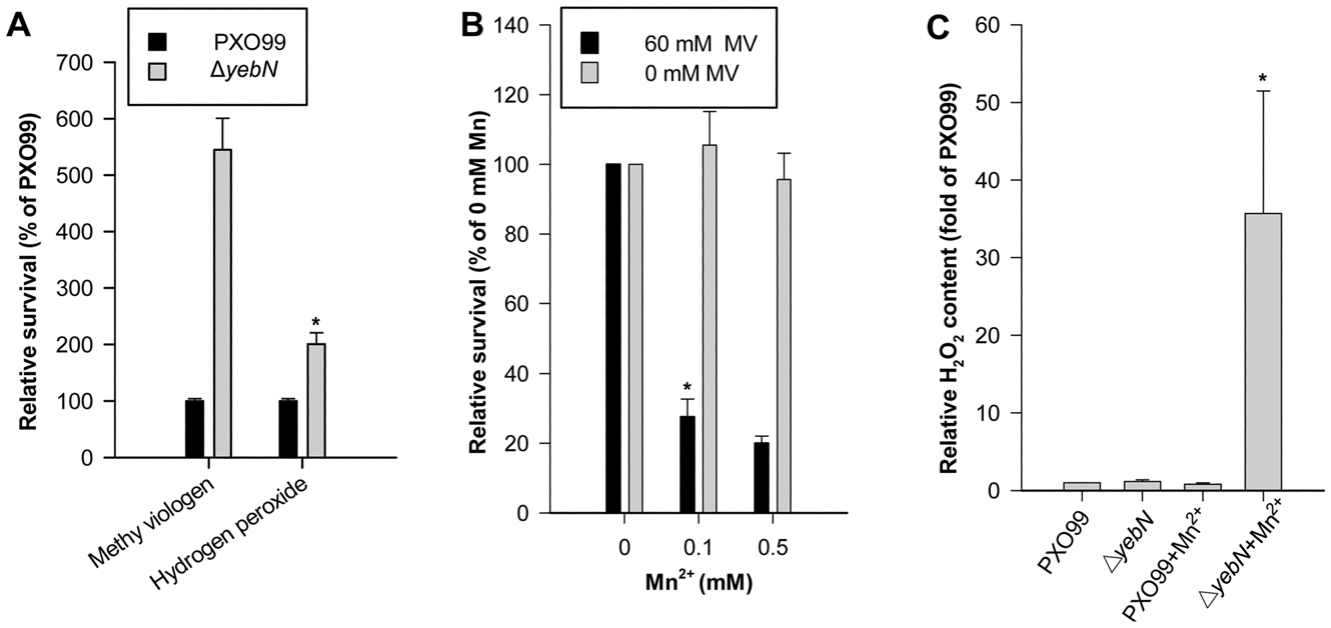
Mutation of *yebN* alters *Xoo* viability under oxidative stress. (A) Strains were grown to an OD of 0.1 then treated with either hydrogen peroxide (10 mM) or methyl viologen (60 mM). Bacterial cfu were calculated immediately before adding oxidants and at 15 min (hydrogen peroxide) or 60 min (methyl viologen, MV) post-treatment using serial dilution estimates and direct counts. (B) Bacteria were treated with manganese (0, 0.1 or 0.5 mM) and methyl viologen (0 or 60 mM), and cfu calculated as described in panel A. Data represent the mean ± SD of the relative survival (% of 0 mM Mn^2+^) from three independent replicates (*P<0.05). (C) Hydrogen peroxide production by the wild type and *yebN* mutant. Bacteria were cultured in PSA media to an OD of 0.5 and then diluted 1∶20 into fresh PSA or PSA supplemented with 0.15 mM MnSO_4_. After growth for 4 additional hours, a 2 ml culture was centrifuged and the pellet re-suspended in sterile PBS. Bacteria were incubated at room temperature for 30 min to allow H_2_O_2_ production. The data shown represents the mean ± SD of the relative H_2_O_2_ content (% of wild type) from three independent replicates (*P<0.05).

Manganese is known to play an important role in resistance to oxidative stress in many bacteria [4], [12], [19]. For example, manganese protects cells against ROS [20] by regulating the activity of superoxide dismutase (SOD) [21]. Consequently, we speculated that *yebN* mutation that increases Mn^2+^ accumulation in *Xoo* cells (Figure 2C) would make cells more tolerant to oxidative stress. Compared to PXO99, Δ*yebN* showed a 5.5 fold increase in cell viability in response to methyl viologen treatment and a 2-fold increase in viability in response to H_2_O_2_ challenge (Figure 6A). However, when bacteria were treated with both Mn^2+^ and methyl viologen, the cell viability of Δ*yebN* was dramatically reduced compared to the wild type (Figure 6B). Because the Mn^2+^ concentration used did not significantly influence bacteria growth (Figure 6B), we speculated that manganese homoeostasis must be critical to ROS production and/or removal, a condition that is normally detrimental to bacterial cells. Consequently, we monitored the production of H_2_O_2_ in the *yebN* and wild type strains in response to Mn^2+^. The PXO99 and *yebN* mutant showed no obvious difference in H_2_O_2_ production in low Mn^2+^ media (Figure 6C). However, at Mn^2+^ concentrations of 0.15 mM, the *yebN* mutant showed a dramatic increase in H_2_O_2_ production (Figure 6C). Thus, in bacteria cells, there might be a threshold level of Mn^2+^ below which the ion functions as an antioxidant. Above this threshold, Mn^2+^ acts as a toxin, inducing excessive ROS production or decreasing ROS scavenger capacity.

### Deletion of *yebN* rendered *Xoo* sensitive to hypo-osmotic shock

**Figure 7:**
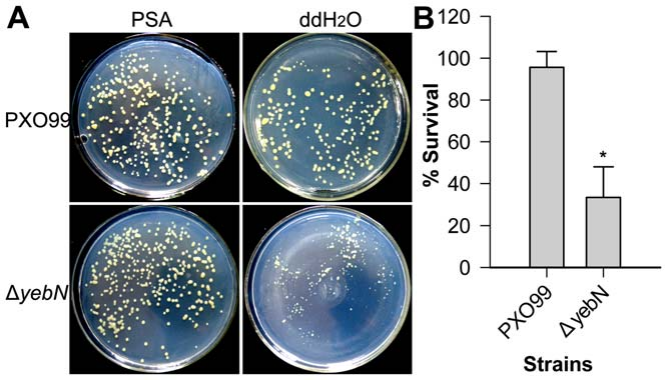
YebN protects bacteria against hypo-osmotic shock. (A) Bacteria were cultured in PSA media to an OD600 of 1.0. A 1 ml culture was centrifuged, the pellet washed twice with PSA, and re-suspended in PSA. 20 µl of a 10-fold serial dilution (left: in PSA, right: in ddH_2_O) of the cells was plated onto PSA plates. (B) Bacteria were cultured in PSA media to an OD600 of 0.5. A 1 ml culture was centrifuged, the pellet re-suspended in PSA and cfu calculated. An additional 1 ml culture was collected as above, washed twice with ddH_2_O and re-suspended in 10 ml ddH_2_O. Bacteria were incubated at room temperature for 60 min and enumerated by serial dilution. The data shown represents the mean ± SD from three independent replicates (*P<0.05).

We found that Δ*yebN* had fewer colony forming units (CFU) and grew more slowly than wild type PXO99 on PSA plates following serial dilution in water, a finding not observed when it had been diluted in culture medium PSA (Figure 7A). Because water is hypo-osmotic and nutrient limited compared to growth medium [22] and we found that both Δ*yebN* and PXO99 exhibited similar growth rates in nutrient limited medium (M4) (Figure 2B), we speculated that YebN could be involved in the hypo-osmotic response. Therefore, we investigated if Δ*yebN* and PXO99 could survive of extreme hypo-osmotic shock. Δ*yebN* suffered ∼70% loss of viability when subjected to a rapid osmotic shock, unlike PXO99 (Figure 7B). These results suggest that YebN protects bacterial cells against hypo-osmotic stress.

### YebN is required for *Xoo* pathogenesis

**Figure 8:**
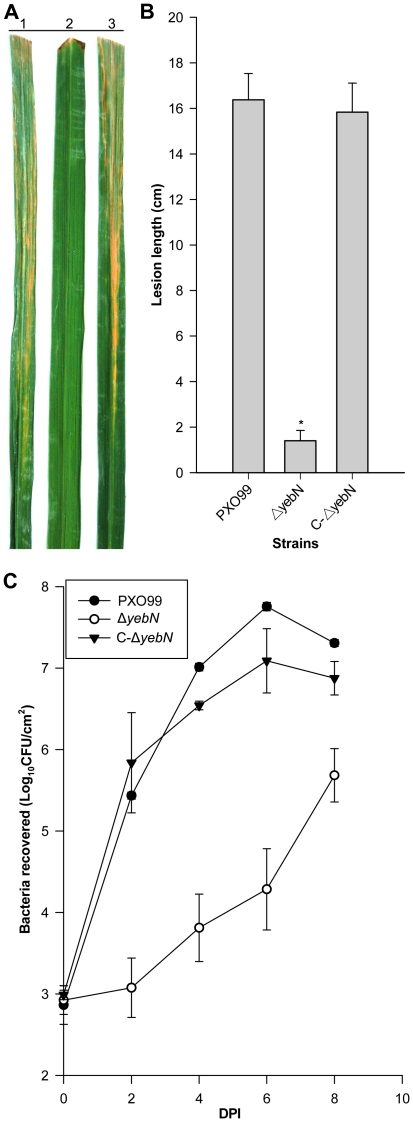
YebN is involved in *X*oo pathogenesis. (A) Lesions on rice leaves inoculated with *X*oo strains. Lane1: PXO99, lane2: Δ*yebN*, lane3: C-Δ*yebN*. 60-day-old susceptible rice cultivars (IR24) were tested. (B) Measurements of the lesion lengths obtained from 20 leaves at 2 weeks post-inoculation. Virulence assays were performed in triplicate and the Mean ± SD were calculated (*P<0.01). (C) Growth of *Xoo* strains in rice leaves. The mean CFU was calculated from three independent experiments using six leaves for each strain.

Most manganese transporters have been reported to play a role in pathogen virulence [10], [12]. Therefore, we infected rice cultivar IR24 with PXO99, Δ*yebN* and the complemented strain C-Δ*yebN* by leaf clipping. Lesion lengths were measured two weeks post-inoculation. We found that the Δ*yebN* lesion was significantly shorter than the lesions formed by PXO99 and the complemented strain, indicating that mutation of *yebN* attenuated *Xoo* virulence (Figure 8A and 8B).

To determine how YebN affects *Xoo* growth in the host, we counted the numbers of bacteria in the infected IR24 rice leaves. In 6 days post-inoculation, the numbers of the wild type and complemented strain were approximately 100-fold higher than those of Δ*yebN* (Figure 8C), suggesting that mutation of *yebN* reduced *Xoo* fitness *in planta.*

## Discussion

In this study, we identified a DUF204 domain containing protein (YebN) that has six transmembrane regions and locates to the cytoplasmic membrane in *Xoo* (Figure 1). Deletion of *yebN* results in sensitivity to exogenous manganese and high-level accumulation of intracellular Mn^2+^ (Figure 2), suggesting that YebN is involved in manganese export. Moreover, *yebN* expression was affected by intracellular manganese ions, but not affected by other metal ions, and positively regulated by manganese via MntR (Figure 5 and S3). Thus, we conclude that YebN is very important for regulating *Xoo* manganese homeostasis. The YebN protein is highly conserved (Figure 1E) and could, therefore, be implicated in manganese export in other bacteria. In *E*. *coli*, we found the *yebN* mutant (JW5830) [23] to be sensitive to exogenous Mn^2+^, but this phenotype could be complemented by the *Xoo* homologue (Figure 4). This finding raises the possibility that YebN could be involved in manganese transport, not only in *Xoo*, but within the wider bacterial kingdom also. Because YebN is conserved across bacteria and rare in eukaryotes, and excessive accumulation of manganese can influence bacterial growth and virulence (Figure 1E, 2 and 8), this DUF204 protein could be an important drug target in bacterial plant pathogens.

YebN does not belong to any transporter family identified to date and sequence similarity searches show that it has no homology to any protein whose function is known. Hence, this study is the first elucidate the function of a DUF204 domain protein. Although Rosch *et al* identified MntE, a member of the CDF inorganic cation transport system that is involved in manganese efflux in *S. pneumoniae* [12], YebN is the first manganese efflux system to be described in a plant bacterial pathogen and could be a member of a newly discovered metal ion transporter family. However, more experiments will be required to prove that YebN is a direct transporter in future work.

To date, very little is known about transition metal ion transport and homeostasis in *Xoo*. Although some studies have been conducted on iron transport and regulation [16], [24], no manganese transporter has been reported in *Xoo* or other *Xanthomonas* species. In the *Xoo* genome, only one homologue of a known manganese transporter gene, *mntH*, was identified [18], but its role in manganese transport is unclear, although it has been shown to be negatively regulated by Mn^2+^ and MntR (Figure S1). Thus, this is the first report that elucidates manganese homeostasis in *Xoo*.

Most bacteria use mechanosensitive channels such as MscL, MscS, MscM, MscK and aquaporins to cope with hypotonic stress [26], [27]. The *Xoo* genome encodes three putative mechanosensitive channel proteins, namely, PXO_03384 (MscS), PXO_00921 (MscS) and PXO_01831 (MscL) [18], whose functions are unknown. Here we have shown that the metal efflux system YebN is involved in *Xoo* hypo-osmotic stress (Figure 7). Prior to this study, there was no direct evidence to suggest that cellular Mn^2+^ could affect the osmotic downshift response in bacteria.

In *Xoo*, manganese possibly functions as a metabolic signal that regulates the osmotic downshift response. In most bacteria, potassium influx is the first response to an osmotic upshift and activates other hyper-osmotic stress responses [28], [29]. Studies have also shown that hyperosmotic stress induces an immediate and transient Ca^2+^ increase in *Saccharomyces cerevisiae and Arabidopsis thaliana* [30], [31]. Another study reported that Ca^2+^ plays an important role in regulating cell volume decrease under hypotonic stress [32]. More recently, Park *et al* found that the *S. enterica* magnesium transporter *mgtA* mRNA levels were enhanced by hyperosmotic shock [33]. These studies demonstrate that bivalent cations can also act as a messenger to active osmotic stress responses.

**Figure 9:**
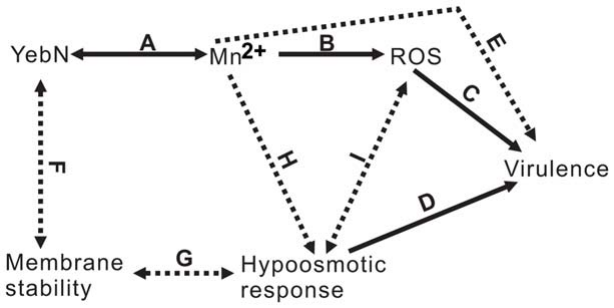
Schematic representation of the roles of YebN in virulence. YebN is vital for manganese accumulation in bacterial cells (A) and possibly regulates cell membrane stability (F) that influences the bacterial hypo-osmotic response (G). Changes in intracellular manganese levels also regulate *yebN* expression (A) and alter its ability to protect bacteria against oxidative stress (B). Manganese and ROS might regulate its capacity to protect bacteria against hypotonic shock (H and I). Hypotonic shock might, in turn, influence ROS production and scavenge (I). Manganese, ROS and hypo-osmosis are all important factors that affect bacterial growth in the host (C, D and E). Solid lines indicate how the results of this study support the model; dashed lines indicate where no direct evidence has been obtained.

Manganese and manganese transporters may regulate hypoosmotic stress by regulating membrane stability (Figure 9F, 9G and 9H). This finding is supported by evidence that membrane-spanning proteins can affect membrane stability [34]. As an integral membrane protein, it is feasible that deletion of *yebN* could affect membrane stability when cells encounter hypotonic shock. However, the G167A mutation that did not affect the YebN subcellular location (Figure S6) resulted in similar phenotypes including manganese sensitivity (Figure 3) and hypotonic shock sensitivity (Figure S7) to the *yebN* deletion mutant, suggesting that absence of YebN might not directly influence the cell membrane stability. Alternatively, manganese has been implicated in regulating the activity of some phosphotransferases that catalyze the biosynthesis of phospholipid and polysaccharide, the indispensable components of cell envelopes [25], [35]. For example, a hemolytic sphingomyelinase from *Pseudomonas* sp. strain TK4 can be activated by Mn^2+^, and the diacylglycerol pyrophosphate phosphatase PgbB of *E*. *coli* is strongly inhibited by Mn^2+^ [36], [37].

YebN may regulate cell volume homeostasis following hyperosmotic stress via ROS scavenging pathways (Figure 9I). Some studies have shown that ROS can modulate swelling-sensitive excitatory amino acid release in cultured astrocytes [38]. Moreover, evidence suggests that ROS can also stimulate the volume-sensitive Cl_2_ current in HeLa and hematoma cells, cultured primary astrocytes and microglia [39]. The *yebN* mutation alters *Xoo* tolerance to exogenous oxidants (Figure 6A and 6B) and causes H_2_O_2_ accumulation (Figure 6C), but its expression was not changed upon hypotonic shock (Figure S8), this would imply that YebN is not synthesized immediately in response to such stress. Hence we speculate that a change in cellular ROS might regulate cell volume-sensitive substrate release upon hypotonic shock, which in turn affects *Xoo* cell viability (Figure 9C and 9D).

It has been shown that manganese and manganese transporters are indispensable for virulence in many bacterial pathogens [1], [4]. Analogous to the situation for other bacterial transporters, inactivation of *yebN* significantly reduced the virulence of *Xoo* (Figure 8), indicating that YebN is a critical virulence determinant. Evidence shows that Mn^2+^ not only acts as a physiological cation for a number of enzymes [1], but also regulates proteins involved in virulence, oxidative stress defense, cellular metabolism, protein synthesis, RNA processing and cell division [40]. This indicates that Mn^2+^ affects pathogen virulence through disparate mechanisms. In the case of *Xoo*, the deletion of *yebN* did not impair bacterial growth in medium (Figure 2B), but significantly reduced bacterial growth *in planta* (Figure 8C). To study how YebN affects *Xoo* pathogenesis, we have tested the effects of *yebN* deletion on the production of virulence factors such as the Type II and Type III secretion systems, extracellular polysaccharide, lipopolysaccharide and swimming/swarming motility, but found no obvious difference between the wild type and *yebN* mutant (data not shown). This suggests that YebN probably regulates *Xoo* virulence through other pathways. Increasingly, evidence suggests that osmotic shock related proteins can function as important virulence factors for certain pathogenic bacteria [41], [42], and it is highly likely that such bacteria will encounter hypo-osmotic stress in their natural habitat [43]. *Xoo* is a vascular pathogen that enters the host through wounds or natural openings such as water pores or hydathodes [44]; thus, maintaining cell stability to hypo-osmotic stress is crucially important to it. Our results show that Δ*yebN* was less viable than the wild type bacteria to hypo-osmotic shock (Figure 7). Therefore, one possible interpretation of the data is that YebN is required for virulence by regulating the hypo-osmotic shock response in the intercellular spaces of the plant (Figure 9D). Other explanations exist, including hitherto unidentified virulence factors regulated by YebN. Firstly, intracellular manganese levels regulate pathogenesis related pilus proteins in a number of bacterial species [12]. Secondly, media may not reproduce the physiological conditions *in planta*. Last of all, manganese might act as a key factor that not only regulates its own homeostasis, but could also be involved in the oxidative response (Figure 9A and 9B). It is noteworthy that deletion of *yebN* results in higher resistance to superoxide and H_2_O_2_ than the wild type strain in low Mn^2+^ concentrations, but confers high sensitivity at high Mn^2+^ concentrations (Figure 6A and 6B). This implies that the *yebN* mutant that might accumulate more Mn^2+^ than the wild type strain (Figure 2C) is more likely to be sensitive to the ROS produced by the host upon infection.

## Materials and Methods

### Bacterial strains and culture conditions

*Xoo* strain PXO99 and its derivatives were grown in liquid or solid PSA medium (10 g/L tryptone; 10 g/L sucrose; 1 g/L L-glutamic acid; 15 g of agar per liter for solid medium) or M4 medium [45] supplemented with 0.1 mM methionine at 28°C. For the GUS activity experiments, sucrose was replaced with glucose (PG medium). *Escherichia coli* cells were cultured in LB (10 g/L tryptone; 5 g/L yeast extract; 10 g/L sodium chloride) at 37°C. Low manganese conditions comprised medium without exogenous manganese. Kanamycin (25 mg/L) and spectinomycin (50 mg/L) were added when appropriate.

### Construction of *Xoo* mutants and complementation strains

*YebN* and *mntR* deletion mutants were created by two exchange steps using the plasmid pK18*mobsacB* [46]. Briefly, upstream and downstream flanking regions ∼ 300 bp from the target gene were PCR amplified and the two PCR fragments ligated into pK18*mobsacB*. The resulting plasmids, pK18MTyebN and pK18MTmntR (Table S1), were introduced into *Xoo* by electroporation. After two rounds of recombination, the open reading frame was deleted from the genomic DNA. Mutants were confirmed by PCR and DNA sequencing.

For construction of the plasmid for complementation of the *yebN* mutant, the full ORF was amplified by PCR using primers c-yebN-F and c-yebN-R (Table S2), then cloned into a broad-host-range vector pHM1 [47]. Point mutation of YebN cytoplasmic domains was introduced by site-directed mutagenesis using the Quick-Change protocol (Stratagene) with primers listed in Table S3. To create a His_6_ –tagged protein in *Xoo* cells, full-length *yebN* was amplified using primers c-yebN-F and yebN-HisTagR which contains the His_6_ coding sequence (Table S2).

### Construction of reporter strains for β-glucuronidase assays

*GusA* was excised from pL6GUS [48] using *Bam*HI and *Eco*RI restriction enzymes and ligated into pK18*mobsacB* yielding plasmid pK18GUS. The promoter of *yebN* was PCR amplified using GUS-5 and GUS-3 primers (Table S2) and ligated into pK18GUS using *Hind*III and *Bam*HI sites, creating pK18UTR-GUS. To obtain pK18mtUTR-GUS (Table S1), a point mutation in the MntR binding site (Figure S2) was introduced by site-directed mutagenesis using the Quick-Change protocol (Stratagene) with primers mntRbsMF and mntRbsMR. pK18UTR-GUS and pK18mtUTR-GUS were integrated into the *mntR* mutant or the PXO99 genome, respectively, by one-step recombination via the *yebN* promoter fragment.

### Cation sensitivity assays

The sensitivity of *Xoo* and *E*. *coli* to metal ions containing plates was tested as described previously [49], [50]. For all plate assays, a fresh single colony was inoculated in 5 ml of medium, and grown aerobically to an OD600 ∼ 0.5. The cells were diluted 10^−1^, 10^−2^, 10^−3^ and 10^−4^ fold in the same media as the assay plates. 2 µl aliquots of cells from each dilution series were inoculated from *left* to *right* onto the assay plates which were then incubated in 28°C prior to photography. Assay medium comprised PSA or LB supplemented with individual metal ions which included different concentrations of calcium chloride, cobalt chloride, magnesium chloride, manganese chloride, iron chloride, nickel chloride and zinc chloride, copper sulfate and cadmium sulfate.

For the growth curves (in liquid media), single colonies were individually inoculated into PSA and grown aerobically to an OD600 of 0.5. Cells were centrifuged, washed twice and resuspended in M4 media before being diluted to an OD600 of 0.086 in 10 ml of M4 medium. Cultures were shaken at 250 r.p.m. at 28°C, and the OD600 measured every 5 hours. M4 medium was supplied with or without MnSO_4_.

### Intracellular manganese concentrations

Intracellular manganese levels in *Xoo* were determined using inductively coupled plasma mass spectrometry (ICP-MS) as previously described [51]. Cells were grown to an OD600 of 0.2 and manganese added to a final concentration of 0.15 mM. After growth to the late exponential phase, *Xoo* cells were harvested by centrifugation, washed twice with cold Tris buffer (50 mM Tris/HCl pH 7.5, 10 mM NaCI, 10 mM KCI) and re-suspended in the same buffer yielding a final concentration of about 40 mg dry mass/ml. Cells were diluted 12 fold in pre-warmed buffer. At specific time points, the cells was harvested by centrifugation and washed with double distilled metal free water to remove salts. Cells were lysed by resuspending pellets in 1 ml of 70% HNO_3_ (Sigma), gently vortexed, then heated to 75°C for 10 min. 9 ml of sterilized Mili-Q water was added to the lysed cells which were mixed by vortexing. Samples were centrifuged at 13,000 *g* for 5 min, and the supernatant analyzed for metal content using ICP-MS. Cfu were enumerated to quantify input and three independent tests were performed.

### Superoxide killing assays

Superoxide killing was performed essentially as previously described [12], [52]. Bacterial cultures were sampled in triplicate and their cell densities adjusted to give equal OD600 readings prior to treatment with H_2_O_2_ (10 mM) for 15 min, or methyl viologen (60 mM) for 60 min. After incubation, aliquots were washed once with fresh medium before being plated onto PSA plates to calculate bacterial numbers.

### Hydrogen peroxide production assays

Bacteria were cultured in PSA media to an OD of 600 of 0.5 and subsequently diluted 1∶20 into fresh PSA or PSA supplemented with 0.15 mM MnSO_4_. After 4 additional hours of growth, 2 ml cultures were centrifuged and re-suspended in sterile PBS. Bacteria were incubated at room temperature for 30 min to allow H_2_O_2_ production and their H_2_O_2_ content measured using a H_2_O_2_ assay kit (Sangon Biotech).

### β-glucuronidase (GUS) reporter assays

Cells were grown in PGA or M4 supplemented with different concentration of Mn^2+^ or other metal ions and harvested by centrifugation. Assays were performed in triplicate using a protocol similar to that described previously [53].

### Electrophorectic mobility shift assays

Full length *mntR* was ligated into pET23b (+) and the resulting plasmid (pET-mntR) transformed into *E*. *coli* BL21 (DE3). MntR expression and purification were conducted according to the His-tag purification manual (QIAGEN). The 95 bp DNA fragment containing the wild type or mutant MntR binding site was amplified using MnBemsaF and MnBemsaR primers. Amplicons were purified using the MinElute Gel Extraction Kit (QIAGEN). Fragments were labeled using T4 polynucleotide kinase (New England BioLabs) and γ-^32^P-ATP. MntR and the labeled probe were incubated for 30 min at room temperature in a 20 µl volume containing EMSA buffer (10 mM HEPES, pH 7.9, 75 mM KCl, 2 mM MgCl_2_, 0.1 mM MnSO_4_, 0.1 mM EDTA, 0.5 mg/ml BSA and 1 mM DTT) after which 5 µl of 5X sample buffer (10 mM HEPES, pH 7.9, 30% glycerol, 0.5% bromophenol blue) was added. 15 µl of the reaction mix was loaded into the individual lanes of a 6% native polyacrylamide gel. The gel was run at 150 V for 2 hours at room temperature, dried and exposed for 4–16 hours to film. The ^32^P-labeled DNA fragment containing the MntR binding site mutation was used as a mutant probe and an unlabeled fragment as a mutant competitor.

### Virulence assay and growth curve *in planta*

Sixty-day-old leaves from the susceptible rice cultivar IR24 were inoculated using the leaf clipping method. *Xoo* strains incubated on PSA plates were suspended at an OD600 of 0.5. 20 expanded upper leaves on the rice were inoculated, and the virulence of the test strains determined 14 days post-inoculation.

To monitor the growth of *Xoo in planta*, previously infected expanded upper leaf sections were assayed every 48 hours. Six leaves were harvested and ground up in 10 mM MgCl_2_. The homogenate was diluted in a 10 mM MgCl_2_ solution and serial dilutions were plated onto PSA plates. The PSA plates were supplemented with antibiotics appropriate for the complemented strain. Bacterial numbers were counted following incubation at 28°C for 48 hours. Assays were independently repeated three times and the number of bacterial populations obtained for each strain calculated from six inoculated leaves at specific time-points.

### Membrane preparations and western blot analysis

To determine the sub-cellular location of YebN, strain C-Δ*yebN-*His was grown in PSA liquid medium to mid-exponential phase. Cells were centrifuged and membrane proteins obtained by ultracentrifugation after cell breakage using a French Pressure cell, as described previously [54]. Protein concentrations were assayed using the BCA assay kit (Genestar). Proteins (2 µg) were separated on 12% Bis-Tris SDS/PAGE gels and western blot analysis conducted using a His-tag antibody in accordance with the manufacturer's instructions (Mathematical Biosciences Institute).

### Real-time quantitative PCR

Total RNA was extracted from PXO99 and Δ*mntR* strains using TRIzol (Invitrogen), and quantified using a Nanodrop ND-100 spectrophotometer (NanoDrop Technologies). Genomic DNA in the RNA samples was removed using RNase-free DNase I (New England BioLabs). cDNA was generated from 5 µg of RNA by using SuperScript III reverse transcriptase (Invitrogen) according to the manufacturer's protocol. Transcript quantification was performed by real-time quantitative PCR (RT-qPCR) using SYBR® *Premix Ex Taq*™ II (Takara Biotechlogy) in an ABI 7500 Sequence Detection System (Applied Biosystems). Results were normalized against the *rpoD* gene. *yebN*, *mntH* and *rpoD* coding regions were detected using the primer pairs yebNqRTF-yebNqRTR, mntHqRTF-mntHqRTR and rpoDqRTF-rpoDqRTR,respectively. A list of the primers used for RT-qPCR is shown in Table S2.

### Data analysis

All assays were repeated at least three times. Data were analyzed using the Student's *t* test and SigmaPlot software. P<0.05 was considered significant. Error bars in graphs indicate standard deviations (SD).

### Nucleotide sequence accession number

The *yebN* and *mntR* sequences of *Xoo* PXO99 were deposited in the GenBank nucleotide sequence databases and the accession numbers are JF680938 and JF680939, respectively.

## Supporting Information

Figure S1 **Manganese negatively regulates** ***mntH*** **expression via transcription factor MntR.** Real time quantitative PCR (qPCR) (A) and RT-PCR (B) analysis of the effect of exogenous manganese on *mntH* expression in wild type *Xoo* (*P<0.05). RNA extraction, RT-PCR and qPCR were conducted as described in Materials and Methods.
(TIF)

Figure S2 **The promoter of** ***yebN*** **comprises an MntR binding site.** Comparison of the putative MntR binding sequences in *yebN* (*Xoo*), *sitABCD* (*S. enterica*) and *mntH* (*S. enterica*) promoters. Bases that are conserved in the putative MntR binding sequences are highlighted. Bases that were modified in the MntR binding site mutant (*MntRbsM*) are shown below the putative binding sequences.
(TIF)

Figure S3 **Deletion of** ***yebN*** **has no visible effects on** ***Xoo*** **growth on plates supplemented with metal ions except Mn^2+^.** The experimental protocol used is the same as that in Figure 2A. The final concentrations of Mn^2+^, Fe^2+^, Zn^2+^, Co^2+^, Cu^2+^, Ni^2+^ and Cd^2+^ were; 1 mM, 5 mM, 0.25 mM, 0.1 mM, 0.1mM, 0.1 mM and 0.01 mM, respectively.
(TIF)

Figure S4 **Amino acid substitutions in** ***Xoo*** **YebN cytoplasmic regions cannot complement** ***E*** **.** ***coli yebN*** **mutation.** (A) The sensitivity of the *E*. *coli yebN* mutant (JW5830) containing *Xoo* YebN with G25A, A26N or G167A substitute to exogenous manganese. The plasmids used were same as Figure 3. The experimental protocol was described in Figure 2A, except *E*. *coli* were cultured in LB medium at 37°C. (B) The effects of amino acid substitutions in *Xoo* YebN cytoplasmic regions on YebN function of resistance to exogenous manganese. All plasmids containing indicated mutations were transformed into JW5830 and growth of transformants was monitored in LB plates (up panel) or LB plates with 1 mM Mn^2+^ (down panel) by streak cultivation.
(TIF)

Figure S5 **YebN regulates its own expression.** *Xoo* strains PXO99 and Δ*yebN* containing the *yebN* promoter *gusA* fusion construct were grown in PSA medium without additional Mn^2+^ and *yebN* expression level (GUS activity) was detected as Figure 4.
(TIF)

Figure S6 **G167A mutant can not affect YebN subcellular location.** The subcellular location of wild type YebN (left) and G167A (right). Up panel, Comassie brilliant blue staining of SDS-PAGE; down panel, western blot using anti-his antibody. An equal amount (2 μg) of protein was loaded in each lane.
(TIF)

Figure S7 **G167A mutant can also cause** ***Xoo*** **sensitive to hypotonic shock.** The *yebN* mutant (Δ*yebN*) containing wild type *yebN* (up panel) or G167A (down panel) mutant in a pHM1 vector was treated as Figure 7A.
(TIF)

Figure S8 **Expression of** ***yebN*** **is not regulated by hypotonic shock.** Bacteria were treated as described in Figure 7B. RNA extraction and real time quantitative PCR (qPCR) were conducted as described in Materials and Methods.
(TIF)

Table S1 **Bacterial strains and plasmid used in this study.**
(DOC)

Table S2 **Oligonucleotide primers for mutant construction, complement,** ***gusA*** **fusion reporter and protein expression used in this study.**
(DOC)

Table S3 **Oligonucleotide primers for point mutation of YebN cytoplasmic regions used in this study.**
(DOC)
