# Supplementary figures and images for: A Novel Manganese Efflux System, YebN, Is Required for Virulence by Xanthomonas oryzae pv. oryzae
Source: PLoS One. 2011 Jul 14;6(7):e21983. doi: 10.1371/journal.pone.0021983 (PMC3136493; doi:10.1371/journal.pone.0021983)

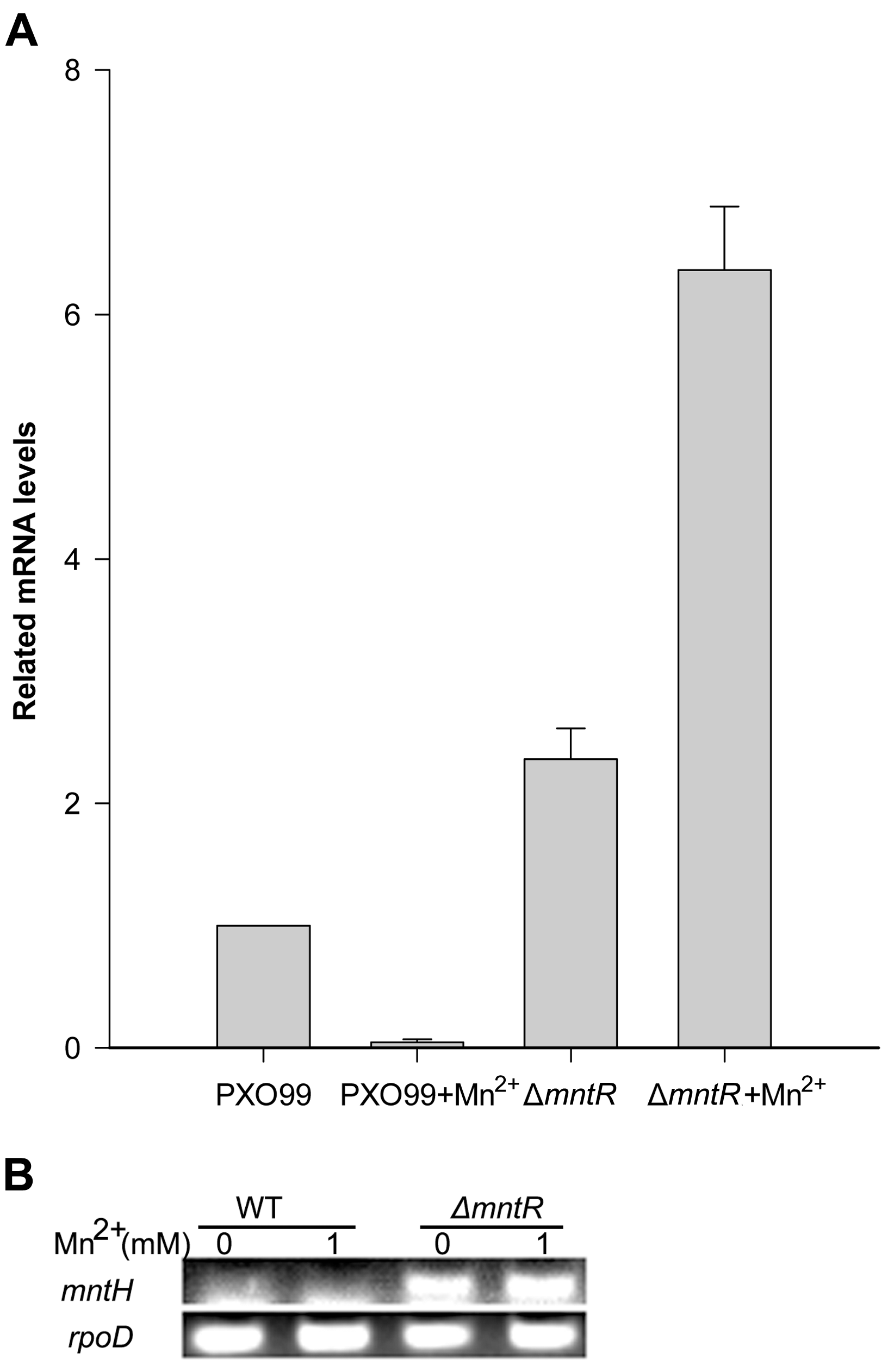

Supplement: Figure S1 — Manganese negatively regulates mntH expression via transcription factor MntR. Real time quantitative PCR (qPCR) (A) and RT-PCR (B) analysis of the effect of exogenous manganese on mntH expression in wild type Xoo (*P<0.05). RNA extraction, RT-PCR and qPCR were conducted as described in Materials and Methods. (TIF) [file pone.0021983.s001.tif]

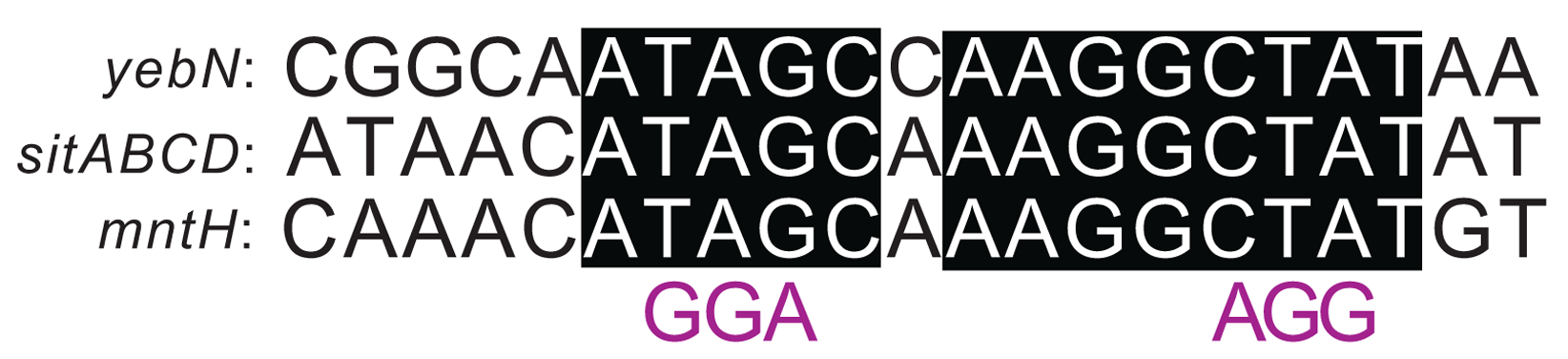

Supplement: Figure S2 — The promoter of yebN comprises an MntR binding site. Comparison of the putative MntR binding sequences in yebN (Xoo), sitABCD (S. enterica) and mntH (S. enterica) promoters. Bases that are conserved in the putative MntR binding sequences are highlighted. Bases that were modified in the MntR binding site mutant (MntRbsM) are shown below the putative binding sequences. (TIF) [file pone.0021983.s002.tif]

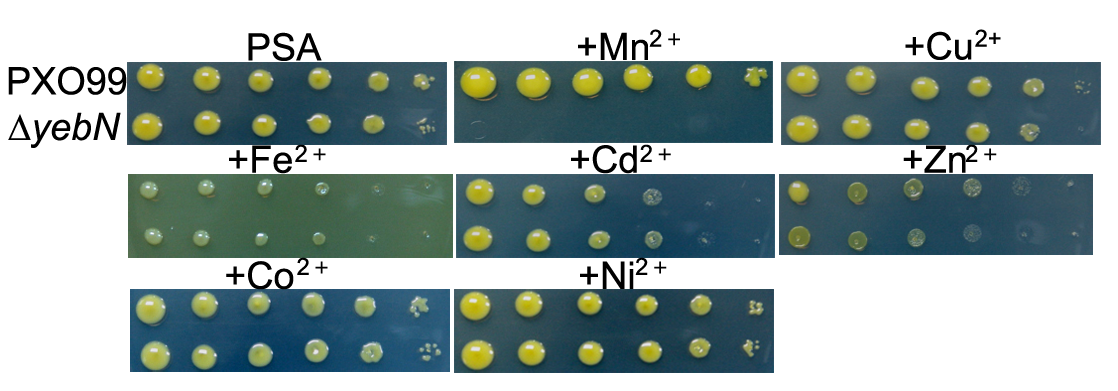

Supplement: Figure S3 — Deletion of yebN has no visible effects on Xoo growth on plates supplemented with metal ions except Mn2+. The experimental protocol used is the same as that in Figure 2A. The final concentrations of Mn2+, Fe2+, Zn2+, Co2+, Cu2+, Ni2+ and Cd2+ were; 1 mM, 5 mM, 0.25 mM, 0.1 mM, 0.1mM, 0.1 mM and 0.01 mM, respectively. (TIF) [file pone.0021983.s003.tif]

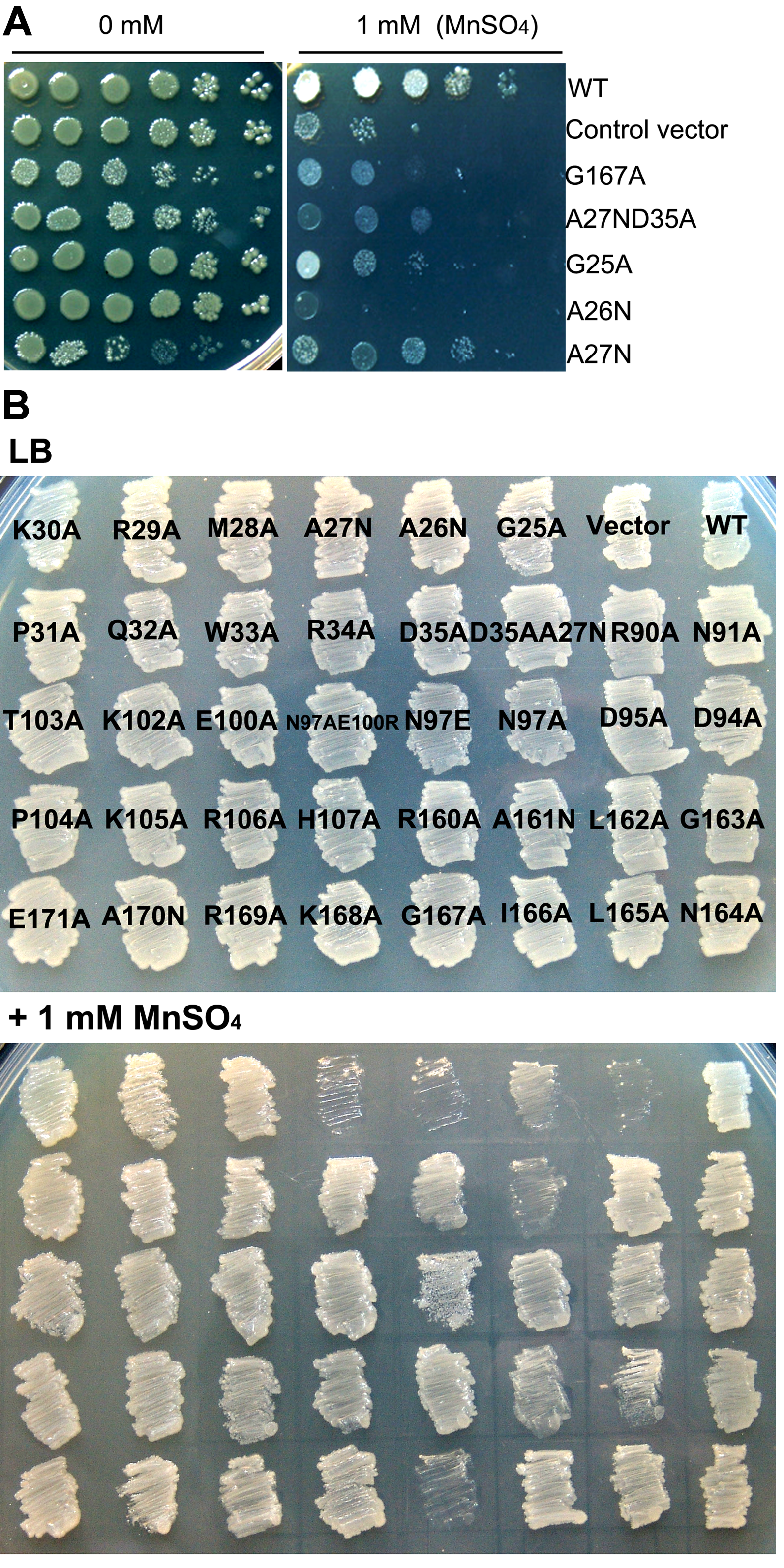

Supplement: Figure S4 — Amino acid substitutions in Xoo YebN cytoplasmic regions cannot complement E . coli yebN mutation. (A) The sensitivity of the E. coli yebN mutant (JW5830) containing Xoo YebN with G25A, A26N or G167A substitute to exogenous manganese. The plasmids used were same as Figure 3. The experimental protocol was described in Figure 2A, except E. coli were cultured in LB medium at 37°C. (B) The effects of amino acid substitutions in Xoo YebN cytoplasmic regions on YebN function of resistance to exogenous manganese. All plasmids containing indicated mutations were transformed into JW5830 and growth of transformants was monitored in LB plates (up panel) or LB plates with 1 mM Mn2+ (down panel) by streak cultivation. (TIF) [file pone.0021983.s004.tif]

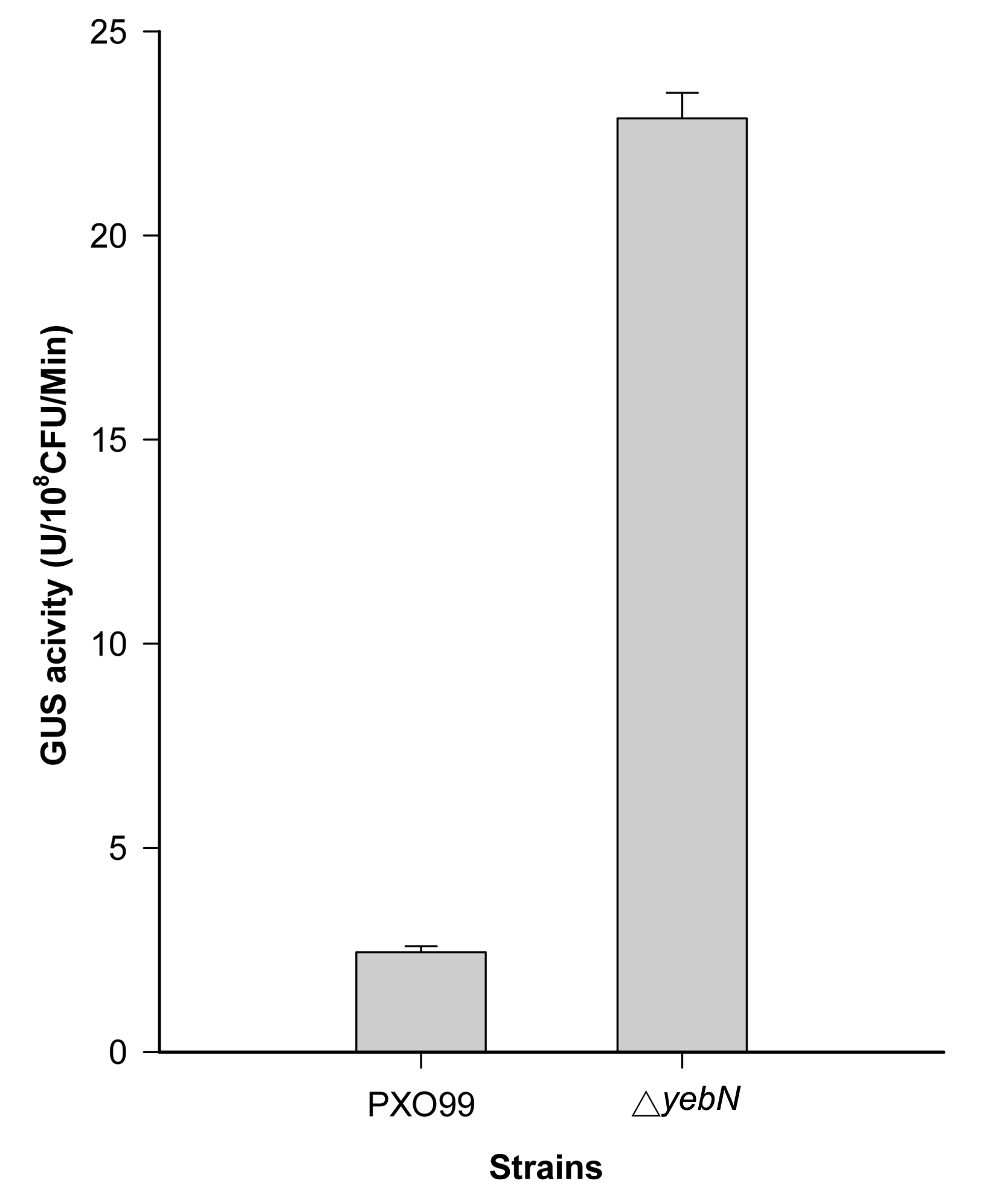

Supplement: Figure S5 — YebN regulates its own expression. Xoo strains PXO99 and ΔyebN containing the yebN promoter gusA fusion construct were grown in PSA medium without additional Mn2+ and yebN expression level (GUS activity) was detected as Figure 4. (TIF) [file pone.0021983.s005.tif]

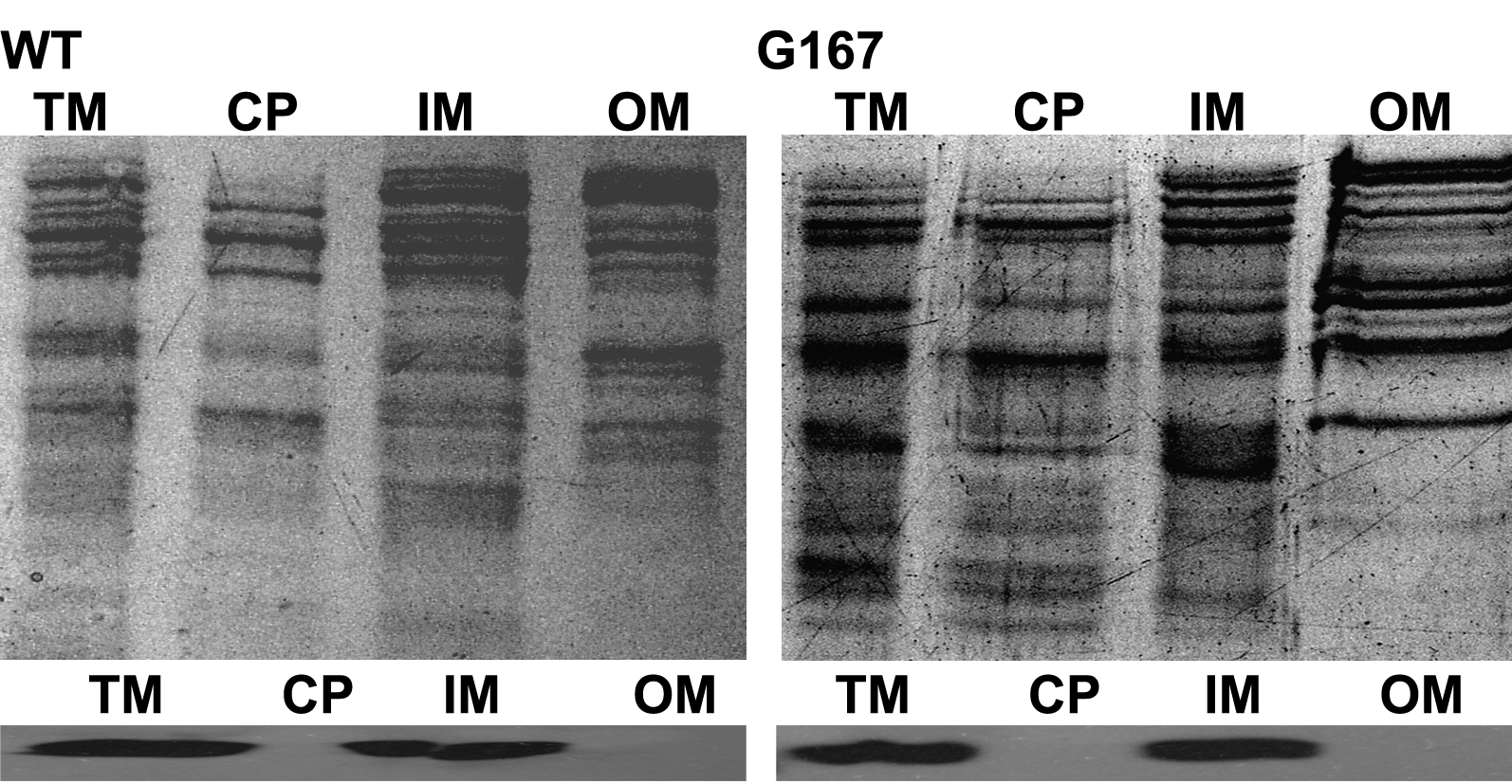

Supplement: Figure S6 — G167A mutant can not affect YebN subcellular location. The subcellular location of wild type YebN (left) and G167A (right). Up panel, Comassie brilliant blue staining of SDS-PAGE; down panel, western blot using anti-his antibody. An equal amount (2 μg) of protein was loaded in each lane. (TIF) [file pone.0021983.s006.tif]

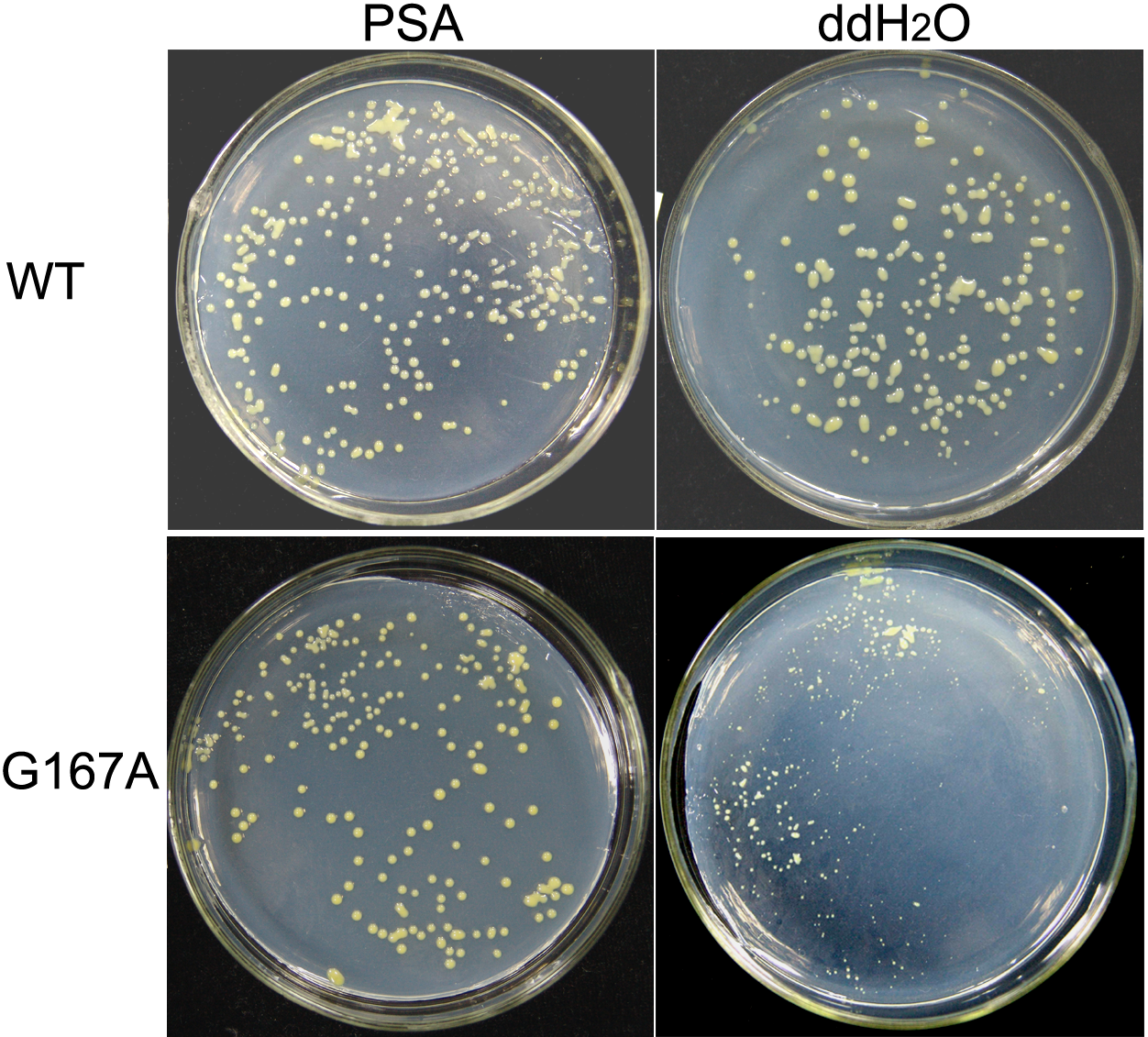

Supplement: Figure S7 — G167A mutant can also cause Xoo sensitive to hypotonic shock. The yebN mutant (ΔyebN) containing wild type yebN (up panel) or G167A (down panel) mutant in a pHM1 vector was treated as Figure 7A. (TIF) [file pone.0021983.s007.tif]

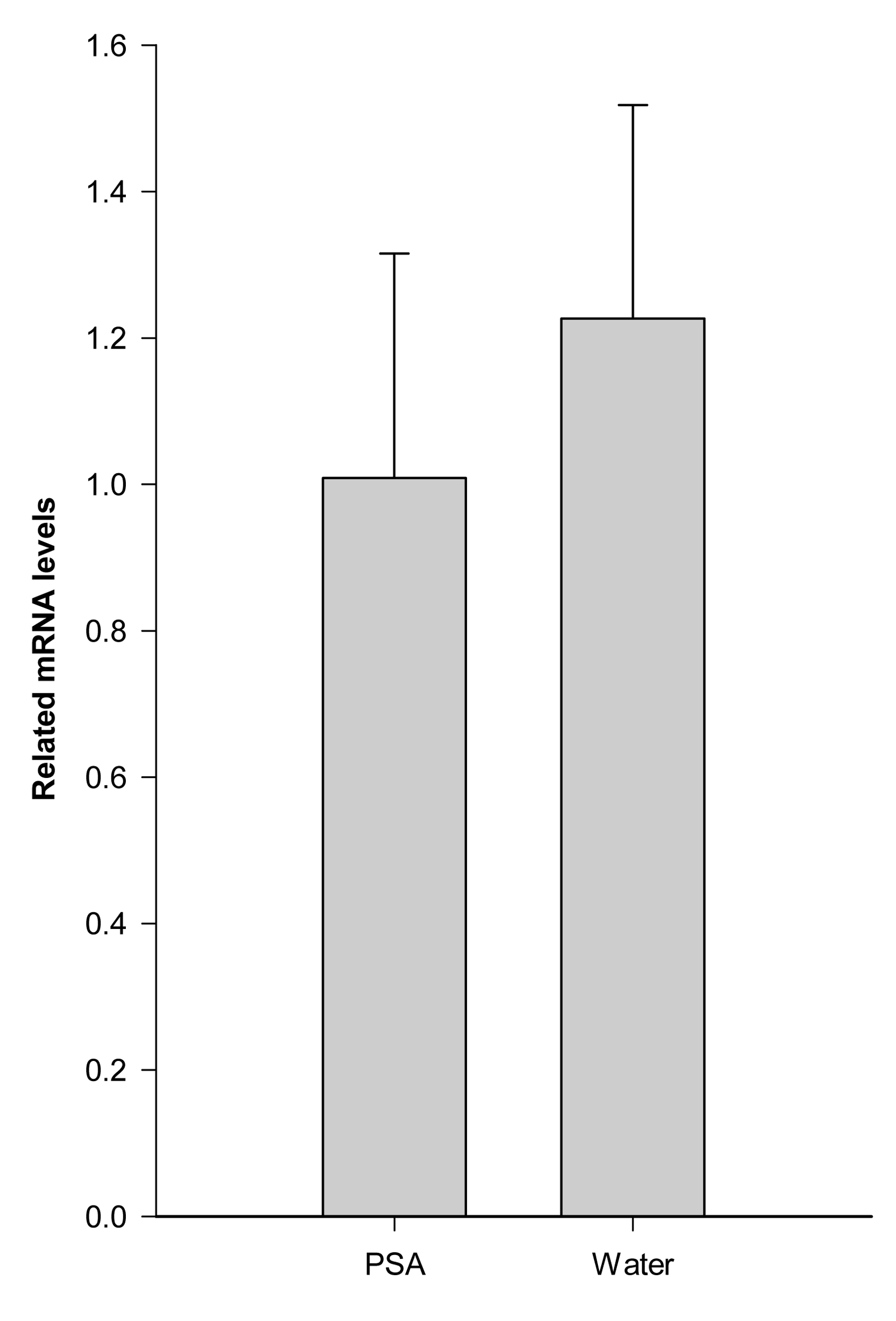

Supplement: Figure S8 — Expression of yebN is not regulated by hypotonic shock. Bacteria were treated as described in Figure 7B. RNA extraction and real time quantitative PCR (qPCR) were conducted as described in Materials and Methods. (TIF) [file pone.0021983.s008.tif]
