# Supplementary material for: A Novel Manganese Efflux System, YebN, Is Required for Virulence by Xanthomonas oryzae pv. oryzae
Source: PLoS One. 2011 Jul 14;6(7):e21983. doi: 10.1371/journal.pone.0021983 (PMC3136493; doi:10.1371/journal.pone.0021983)
Supplement: Table S1 — Bacterial strains and plasmid used in this study. (DOC) [file pone.0021983.s009.doc]

**Table S1. Bacterial strains and plasmid used in this study**

| Name | Relevant genotype or description | Reference |
| --- | --- | --- |
| Strains |  |  |
| *Xoo* |  |  |
| PXO99 | Philippine race 6 (PR6) strain, avirulent on Xa21 rice plants, wild type | [1] |
| Δ*yebN* | Full length deletion of *yebN* | This work |
| Δ*mntR* | Full length deletion of *mntR* | This work |
| C-Δ*yebN* | PXO99 containing pHM-yebN which contains full length *yebN* gene | This work |
| C-Δ*yebN*-His | PXO99 containing pHM-yebN-his | This work |
| WT-GUS | Integration of pK18UTR-GUS into PXO99 genome via the *yebN* promoter sequence | This work |
| Δ*mntR*-GUS | Integration of pK18UTR-GUS into Δ*mntR* genome via the *yebN* promoter sequence | This work |
| WT-mtGUS | Integration of pK18mtUTR-GUS into PXO99 genome via the *yebN* promoter sequence | This work |
| Δ*mntR*-mtGUS | Integration of pK18mtUTR-GUS into *ΔmntR* genome via the *yebN* promoter sequence | This work |
| E. coli |  |  |
| DH10B | F−mcrA Δ(mrr− hsd RMS− mcrBC) Φ80dlacZΔM15 ΔlacX74 endA1 recA1deoR Δ(ara, leu)7697 araD139 galU galK nupG rpsL thi pro hsdR− hsd+recA RP4-2-TcΔMu-Tn7 | [2] |
| BL21-AI | Chromosome Contains T7 RNA polymerase gene (T7 RNAP) under control of the arabinose-inducible araBAD promoter | Invitrogen |
| Plasmids |  |  |
| pGEM-T easy | PCR cloning vector, Amp^R^ | Promega LTD, USA |
| pK18*mobSacB* | Suicide plasmid in *Xanthomonas*, Km^R^ | [3] |
| pK18MTyebN | Sucide construct for in-frame deletion of full length *yebN*, Km^R^ | This work |
| pK18MTmntR | Sucide construct for in-frame deletion of full length *mntR*, Km^R^ | This work |
| pK18GUS | pK18mobSacB containing promoterless *gusA*, Km^R^ | This work |
| pK18UTR-GUS | pK18GUS with *yebN* promoter sequence, Km^R^ | This work |
| pK18mtUTR-GUS | MntR binding site mutation in pK18UTR-GUS | This work |
| pHMI | Broad host range cosmid vector (~13.3kb), Sp^R^ | [4] |
| pHM-yebN | pHMI containing full sequence of *yebN*, Sp^R^ | This work |
| pHM-yebN-his | pHMI containing full sequence of *yebN* with 6X his tag sequence, Sp^R^ | This work |
| pET23b(+) | Expression vector carrying an N-terminal T7•Tag sequence plus an optional C-terminal His•Tag  Sequence, Amp^R^ | Novagen |
| pET-mntR | pET23b(+) containing full length of *mntR*, Amp^R^ | This work |

Amp^R^, Sp^R^ and Km^R^ indicate resistance to ampicillin, spectinomycin and Kanamycin, respectively

1. Hopkins CM, White FF, Choi SH, Guo A, Leach JE (1992) Identification of a family of avirulence genes from *Xanthomonas oryzae* pv. *oryzae*. Mol Plant Microbe Interact 5: 451-459.

2. Simon R, Priefer U, Puhler A (1983) A broad host range mobilization system for *in vivo* genetic engineering: transposon mutagenesis in gram negative bacteria. Bio/Technology 1: 784-791.

3. Schafer A, Tauch A, Jager W, Kalinowski J, Thierbach G, et al. (1994) Small mobilizable multi-purpose cloning vectors derived from the *Escherichia coli* plasmids pK18 and pK19: selection of defined deletions in the chromosome of *Corynebacterium glutamicum*. Gene 145: 69-73.

4. Huynh TV, Dahlbeck D, Staskawicz BJ (1989) Bacterial blight of soybean: regulation of a pathogen gene determining host cultivar specificity. Science 245: 1374-1377.
