# Supplementary material for: A Novel Manganese Efflux System, YebN, Is Required for Virulence by Xanthomonas oryzae pv. oryzae
Source: PLoS One. 2011 Jul 14;6(7):e21983. doi: 10.1371/journal.pone.0021983 (PMC3136493; doi:10.1371/journal.pone.0021983)
Supplement: Table S2 — Oligonucleotide primers for mutant construction, complement, gusA fusion reporter and protein expression used in this study. (DOC) [file pone.0021983.s010.doc]

**Table S2. Oligonucleotide primers for mutant construction, complement, *gusA* fusion reporter and protein expression used in this study***

| Name | 5’-3’ |
| --- | --- |
| yebN-ddFF | CAGTCTAGATCAGACTGACCTTCGCGTAAGG |
| yebN-ddFR | CATGGTACCGGCAAACGCGTCGGTGGACATC |
| yebN-ddRF | CGAGGTACCGGATTGATTCTGGTGATCGTC |
| yebN-ddRR | ACGAAGCTTTTGAGTGCGCATCACGATGC |
| mntR-ddFF | GTCAAGCTTTAGGCTACCGGTGGCAGTGG |
| mntR-ddFR | GAAGGTACCCACCTGACGGAAGCTCTCCA |
| mntR-ddRF | GACGGTACCCATGTCAGTGAGGCGACGGT |
| mntR-ddRR | GCGTCTAGATCAGGCGGTGATTGGACAGG |
| c-yebN-F | GCAAGCTTATGTCTCCTTTTTCCATTGTG |
| c-yebN-R | TAGGTACCCACGCGGCATCCATTACAC |
| GUS-5 | TAGAAGCTTTAGCCCAGGCTAGCCGCGGACATG |
| GUS-3 | CAGGATCCAACGCGTCGGTGGACATCG |
| mntRbsMF | GTCTCGCACCCGGCAAGGACCAAGGAGGTAAAGATCAGACTGAC |
| mntRbsMR | GTCAGTCTGATCTTTACCTCCTTGGTCCTTGCCGGGTGCGAGAC |
| yebNqRTF | ATGCCAACGATGCGGAG |
| yebNqRTR | GAGAAACGCCAGGCTGA |
| mntHqRTF | GCGCGCTGGAAGCATTCGTG |
| mntHqETR | ACCTGCGCACGCGGAATGAA |
| rpoDqRTF | CGACAACACCACCAACATCAATCT |
| rpoDqRTR | AACTGCTTACCGACCTCTTCCAAC |
| yebN-HisTagR | GGGAATTCTTAGTGGTGATGGTGATGATGCGTGGCGGCGCCCAGGTGTTC |
| mntR-ex-F | GTGGATCCGGTGGGCAAGAGCGAAAAGATG |
| mntR-ex-R | TGAAGCTTCGTGGCCGAACTGCCCTGG |
| MnBemsaF | CTCAGACAACAACAGCCTGTC |
| MnBemsaR | CCGGTTTTCGCGCTTCTTGC |

*All primers were designed based on PXO99^A^ sequence; Restriction enzyme sites are underlined.
