# Supplementary material for: A Novel Manganese Efflux System, YebN, Is Required for Virulence by Xanthomonas oryzae pv. oryzae
Source: PLoS One. 2011 Jul 14;6(7):e21983. doi: 10.1371/journal.pone.0021983 (PMC3136493; doi:10.1371/journal.pone.0021983)
Supplement: Table S3 — Oligonucleotide primers for point mutation of YebN cytoplasmic regions used in this study. (DOC) [file pone.0021983.s011.doc]

**Table S3. Oligonucleotide primers for point mutation of YebN cytoplasmic regions used in this study**

| Name | 5’-3’ |
| --- | --- |
| G25AF | CGGCGATCGGCAAGGCCGCGGCGATGCGCAAAC |
| G25AR | GTTTGCGCATCGCCGCGGCCTTGCCGATCGCCG |
| A26NF | GGCGATCGGCAAGGGCAACGCGATGCGCAAACCGCA |
| A26NR | TGCGGTTTGCGCATCGCGTTGCCCTTGCCGATCGCC |
| A27NF | GATCGGCAAGGGCGCGAATATGCGCAAACCGCAGTG |
| A27NR | CACTGCGGTTTGCGCATATTCGCGCCCTTGCCGATC |
| M28AF | TCGGCAAGGGCGCGGCGGCGCGCAAACCGCAGTG |
| M28AR | CACTGCGGTTTGCGCGCCGCCGCGCCCTTGCCGA |
| R29AF | CAAGGGCGCGGCGATGGCCAAACCGCAGTGGCGC |
| R29AR | GCGCCACTGCGGTTTGGCCATCGCCGCGCCCTTG |
| K30AF | AGGGCGCGGCGATGCGCGCGCCGCAGTGGCGCGATG |
| K30AR | CATCGCGCCACTGCGGCGCGCGCATCGCCGCGCCCT |
| P31AF | CGCGGCGATGCGCAAAGCGCAGTGGCGCGATG |
| P31AR | CATCGCGCCACTGCGCTTTGCGCATCGCCGCG |
| Q32AF | GGCGATGCGCAAACCGGCGTGGCGCGATGCGCTG |
| Q32AR | CAGCGCATCGCGCCACGCCGGTTTGCGCATCGCC |
| W33AF | GATGCGCAAACCGCAGGCGCGCGATGCGCTGCG |
| W33AR | CGCAGCGCATCGCGCGCCTGCGGTTTGCGCATC |
| R34AF | CGCAAACCGCAGTGGGCCGATGCGCTGCGCGCC |
| R34AR | GGCGCGCAGCGCATCGGCCCACTGCGGTTTGCG |
| D35AF | CAAACCGCAGTGGCGCGCCGCGCTGCGCGCCGGT |
| D35AR | ACCGGCGCGCAGCGCGGCGCGCCACTGCGGTTTG |
| R90AF | GATGATCGCCGGCCTGGCCAATGGGCCGGACGATG |
| R90AR | CATCGTCCGGCCCATTGGCCAGGCCGGCGATCATC |
| N91AF | GATCGCCGGCCTGCGCGCGGGGCCGGACGATGC |
| N91AR | GCATCGTCCGGCCCCGCGCGCAGGCCGGCGATC |
| D94AF | CTGCGCAATGGGCCGGCCGATGCCAACGATGCG |
| D94AR | CGCATCGTTGGCATCGGCCGGCCCATTGCGCAG |
| D95AF | CGCAATGGGCCGGACGCCGCCAACGATGCGGAG |
| D95AR | CTCCGCATCGTTGGCGGCGTCCGGCCCATTGCG |
| N97EF | TGGGCCGGACGATGCCGAGGATGCGGAGGCCAAGAC |
| N97ER | GTCTTGGCCTCCGCATCCTCGGCATCGTCCGGCCCA |
| N97AF | TGGGCCGGACGATGCCGCGGATGCGGAGGCCAAGAC |
| N97AR | GTCTTGGCCTCCGCATCCGCGGCATCGTCCGGCCCA |
| N97AE100AF | TGGGCCGGACGATGCCGCGGATGCGGCGGCCAAGACGCCGAAAC |
| N97AE100AR | GTTTCGGCGTCTTGGCCGCCGCATCCGCGGCATCGTCCGGCCCA |
| E100AF | GATGCCAACGATGCGGCGGCCAAGACGCCGAAAC |
| E100AR | GTTTCGGCGTCTTGGCCGCCGCATCGTTGGCATC |
| K102AF | CAACGATGCGGAGGCCGCGACGCCGAAACGGCATG |
| K102AR | CATGCCGTTTCGGCGTCGCGGCCTCCGCATCGTTG |
| T103AF | GATGCGGAGGCCAAGGCGCCGAAACGGCATGGC |
| T103AR | GCCATGCCGTTTCGGCGCCTTGGCCTCCGCATC |
| P104AF | TGCGGAGGCCAAGACGGCGAAACGGCATGGCTTG |
| P104AR | CAAGCCATGCCGTTTCGCCGTCTTGGCCTCCGCA |
| K105AF | GAGGCCAAGACGCCGGCCCGGCATGGCTTGCTG |
| K105AR | CAGCAAGCCATGCCGGGCCGGCGTCTTGGCCTC |
| R106AF | AGGCCAAGACGCCGAAAGCGCATGGCTTGCTGGGC |
| R106AR | GCCCAGCAAGCCATGCGCTTTCGGCGTCTTGGCCT |
| H107AF | CAAGACGCCGAAACGGGCGGGCTTGCTGGGCTTG |
| H107AR | CAAGCCCAGCAAGCCCGCCCGTTTCGGCGTCTTG |
| R160AF | CAGGCGTGATGCTGGGCGCCGCGCTCGGCAATCTG |
| R160AR | CAGATTGCCGAGCGCGGCGCCCAGCATCACGCCTG |
| A161NF | CGTGATGCTGGGCCGCAATCTCGGCAATCTGATCG |
| A161NR | CGATCAGATTGCCGAGATTGCGGCCCAGCATCACG |
| L162AF | GATGCTGGGCCGCGCGGCCGGCAATCTGATCGGCA |
| L162AR | TGCCGATCAGATTGCCGGCCGCGCGGCCCAGCATC |
| G163AF | CTGGGCCGCGCGCTCGCCAATCTGATCGGCAAG |
| G163AR | CTTGCCGATCAGATTGGCGAGCGCGCGGCCCAG |
| N164AF | CTGGGCCGCGCGCTCGGCGCGCTGATCGGCAAGCGCG |
| N164AR | CGCGCTTGCCGATCAGCGCGCCGAGCGCGCGGCCCAG |
| L165AF | CCGCGCGCTCGGCAATGCGATCGGCAAGCGCGCC |
| L165AR | GGCGCGCTTGCCGATCGCATTGCCGAGCGCGCGG |
| I166AF | CGCGCTCGGCAATCTGGCCGGCAAGCGCGCCGAA |
| I166AR | TTCGGCGCGCTTGCCGGCCAGATTGCCGAGCGCG |
| G167AF | GCTCGGCAATCTGATCGCCAAGCGCGCCGAAATTC |
| G167AR | GAATTTCGGCGCGCTTGGCGATCAGATTGCCGAGC |
| K168AF | GGCAATCTGATCGGCGCGCGCGCCGAAATTCTG |
| K168AR | CAGAATTTCGGCGCGCGCGCCGATCAGATTGCC |
| R169AF | CAATCTGATCGGCAAGGCCGCCGAAATTCTGGGTG |
| R169AR | CACCCAGAATTTCGGCGGCCTTGCCGATCAGATTG |
| A170NF | CTGATCGGCAAGCGCAACGAAATTCTGGGTGGAT |
| A170NR | ATCCACCCAGAATTTCGTTGCGCTTGCCGATCAG |
| E171AF | GATCGGCAAGCGCGCCGCCATTCTGGGTGGATTG |
| E171AR | CAATCCACCCAGAATGGCGGCGCGCTTGCCGATC |
